# Clinical Significance of Different Profiles of anti-Ro Antibodies in Connective Tissue Diseases

**DOI:** 10.1155/2023/9195157

**Published:** 2023-01-25

**Authors:** Hai-Tao Yang, Xiao-Ping Hong, Jie-Wen Guo, Xiao-Ling Zhong, Rui Liao, Cui-Lian Liu, Li-Xiong Liu, Kai Li, Yu-Lan Chen, Dong-Zhou Liu

**Affiliations:** ^1^Department of Rheumatology and Immunology, Shenzhen People's Hospital (The Second Clinical Medical College, Jinan University; The First Affiliated Hospital, Southern University of Science and Technology), Shenzhen, Guangdong, China; ^2^The Second Clinical Medical College, Jinan University (Shenzhen People's Hospital), Shenzhen, Guangdong, China; ^3^Department of Radiology, Shenzhen People's Hospital (The Second Clinical Medical College, Jinan University; The First Affiliated Hospital, Southern University of Science and Technology), Shenzhen, Guangdong, China

## Abstract

**Objective:**

Anti-Ro60 and anti-Ro52 antibodies are associated with different connective tissue diseases (CTDs). However, the clinical significance of anti-Ro antibodies is not always consistent among different global regions. The aim of this study was to investigate the clinical characteristics of patients with anti-Ro antibodies.

**Methods:**

A total of 1596 inpatients with anti-Ro antibodies were included in the study. Demographic, clinical, and serological data were compared between individuals with different profiles of anti-Ro antibodies: patients with anti-Ro52 antibodies alone, patients with anti-Ro60 antibodies alone, and patients with combined anti-Ro52 and anti-Ro60 antibodies.

**Results:**

Of the 1596 patients, 1362 (85.3%) were female, the mean age was 45.5 years, and systemic lupus erythematosus (SLE) (46.0%) and Sjogren's syndrome (SS) (19.0%) were the most common CTD diagnoses. Among the patients with anti-Ro52 antibodies alone, idiopathic inflammatory myopathy (18.8%) and SLE (17.6%) were the most common CTD diagnoses. The coexistent autoantibodies of this group were significantly lower compared with those of the other two groups, while the presence of anti-Jo1 antibodies were significantly higher compared with those of the other two groups (3.7% vs. 0.6% vs. 1.9%, *p* = 0.029). In addition, the patients with isolated anti-Ro52 antibodies were more likely to suffer from interstitial lung disease (35.5% vs. 11.3% vs. 13.7%, *p* < 10^−4^) and pulmonary arterial hypertension (10.1% vs. 5.3% vs. 3.6%, *p* = 0.001) compared with the other two groups of patients. Compared with patients with isolated anti-Ro52 or anti-Ro60 antibodies, the patients with combined anti-Ro52 and anti-Ro60 antibodies were more likely to suffer from xerophthalmia and xerostomia. Furthermore, hypocomplementemia, hyperglobulinemia, and proteinuria were particularly prevalent in patients with anti-Ro60 antibodies.

**Conclusion:**

Different profiles of anti-Ro antibodies were significantly associated with clinical phenotypic features in CTDs, indicating the potential diagnostic and prognostic value of these antibodies in clinical practice.

## 1. Introduction

Autoimmune diseases are defined as pathological manifestations related to immune responses against autoantigens. Autoimmune diseases can be divided into organ-specific diseases and systemic diseases, which are characterized by the involvement of multiple organs and the presence of autoantibodies [1]. Connective tissue diseases (CTDs) are a heterogeneous group of systemic autoimmune disorders, including rheumatoid arthritis (RA), systemic lupus erythematosus (SLE), Sjögren syndrome (SS), systemic sclerosis (SSc), inflammatory myopathies (IM), and mixed connective tissue diseases (MCTD).

Two structurally unrelated proteins, Ro60 and Ro52, were recognized by sera from patients with anti-Ro antibodies [2, 3], which have subsequently been found to be associated with various CTDs, such as SS and SLE [4]. Ro60, an RNA-binding protein with a molecular weight of 60 kDa, acts as a quality checkpoint for defective RNAs [5]. Anti-Ro60 antibodies are a mandatory criterion for the classification of patients with SS, especially in patients with a negative labial gland biopsy [6]. Ro52, also known as tripartite motif 21(TRIM21), is a 52-kDa E3 ubiquitin ligase [7]. Anti-Ro52 antibodies are positively associated with glandular dysfunction, parotid enlargement, hypergammaglobulinemia, and rheumatoid factor (RF) positivity in patients with SS [8]. In addition, anti-Ro52 is one of the most common antibodies and it usually cooccurs with anti-Jo-1 antibodies in patients with IM [9]. Compared with those without interstitial lung disease (ILD), patients with ILD have a higher prevalence of anti-Ro52 antibodies in SS, SSc, and MCTD [10–12]. Furthermore, although anti-Ro52 antibodies are one of the most frequent antibodies in CTDs [13], they are also common in some non-CTDs, such as malignancies and infections [14, 15]. Overall, anti-Ro antibodies have been proven to be implicated in different diseases. Nevertheless, most previous studies have merely focused on the clinical significance of anti-Ro antibodies in several specific diseases, such as SS, ILD, and IM. In addition, although Zampeli et al. and Robbins et al. have, respectively, demonstrated the disease distribution and coexistent antibody profiles in patients with different anti-Ro profiles, the results were derived from relatively small cohorts [16, 17]. Here, a large-scale single-center study was therefore performed to comprehensively explore the clinical relevance and the disease phenotypes of patients with different anti-Ro antibody profiles.

## 2. Methods

### 2.1. Study Population

Consecutive inpatients that were positive for anti-Ro52 or anti-Ro60 antibodies were retrospectively recruited from Shenzhen People's Hospital between September 2015 and March 2020. Inclusion criteria for the study also included: (1) patients aged ≥ 18 years; and (2) individuals with complete medical records. Patients diagnosed with CTDs, including SLE [18–20], SS [6, 21, 22], RA [23, 24], SSc [25, 26], undifferentiated connective tissue diseases (UCTD) [27], and IM [28], fulfilled the international criteria for classification. Other diagnoses, such as systemic vasculitis [29], antiphospholipid syndrome [30], spondylarthritis [31–33], and MCTD [34], were defined according to the recognized classification criteria. Patients who concurrently fulfilled the classification criteria for two or more types of systemic CTDs (SLE, RA, IM, and SSc) were diagnosed with overlap syndromes. Patients with systemic CTDs who concurrently met the criteria for SS were defined as secondary SS to those systemic CTDs rather than overlap syndromes, and were classified into the corresponding CTD groups. The study was approved by the Medical Ethics Committee of Shenzhen People's Hospital (identifier: LL-KY-2022158-01).

### 2.2. Autoantibody Detection

Serum autoantibodies to extractable cellular antigens (ENAs) were detected using a commercially available line immunoblot assay (Yahuilong Biotech Co., Ltd, Shenzhen, China), including 17 different antibodies to ENAs: anti-nucleosome, anti-double-stranded DNA (ds-DNA), anti-histone, anti-SmD1, anti-proliferating cell nuclear antigen (PCNA), anti-ribosome P0 protein anti-P0), anti-Ro60, anti-Ro52, anti-La, anti-centromere B (CENP-B), anti-topoisomerase I (Scl-70), anti-U1-small nuclear ribonucleoprotein (U1-snRNP), anti-mitochondrial antibody M2 subtype (AMA-M2), anti-Jo-1, anti-polymyositis/scleroderma (PM-Scl), anti-Mi-2, and anti-Ku antibodies. Anti-nuclear antibodies (ANA) were detected by indirect immunofluorescence (EUROIMMUN, Lübeck, Germany) and/or chemiluminescence assays (Yahuilong Biotech Co., Ltd). Antibodies to cardiolipin immunoglobulin (IgM/IgG), *β*2-glycoprotein 1 (*β*2-GP1) IgG, cyclic citrullinated peptide (anti-CCP), and rheumatoid arthritis 33 (RA33) were evaluated by enzyme-linked immunosorbent assay (ELISA) (Yahuilong Biotech Co., Ltd). Rheumatoid factor (RF) was detected by turbidimetric inhibition immunoassay **(**Genrui Biotech Inc, Shenzhen, China**).**

### 2.3. Clinical and Laboratory Data Collection

Baseline clinical data, including demographics and clinical manifestations, were collected from the medical records of the inpatients at the time of diagnosis. Baseline laboratory data comprised complete blood count, serum complement C3 (normal range: 0.8–1.81 g/L) and C4 levels (normal range: 0.15–0.57 g/L), IgG levels (normal range: 8–20 g/L), estimated glomerular filtration rate (eGFR), serum albumin, and levels of erythrocyte sedimentation rate (ESR) and C-reaction protein (CRP).

In addition to constitutional symptoms, organ involvements—predominantly mucocutaneous, musculoskeletal, renal, hematological, cardiovascular, pulmonary, and neuropsychiatric involvements—were defined according to individuals' symptoms and laboratory and radiologic data. The definition of each organ involvement was as previously described [35].

### 2.4. Statistical Analysis

Statistical analysis was performed using SPSS 21.0 (IBM Corp., Armonk, NY, USA). Descriptive data are presented as mean ± standard deviation for continuous variables, and as frequencies and percentages (%) for categorical variables. Differences in continuous variables between groups were compared using Student's *t*-test and one-way analysis of variance (ANOVA) or Mann–Whitney test when appropriate, while categorical variables were compared using *χ*2 test or Fisher's exact test. In multiple comparisons, the homogeneity of variance used the Bonferroni method, while missing variance used the Games–Howell method. *p* values were adjusted for multiple testing. All significance tests were two-tailed and *p* < 0.05 were considered significant.

## 3. Results

### 3.1. Demographic Characteristics

**Figure 1:**
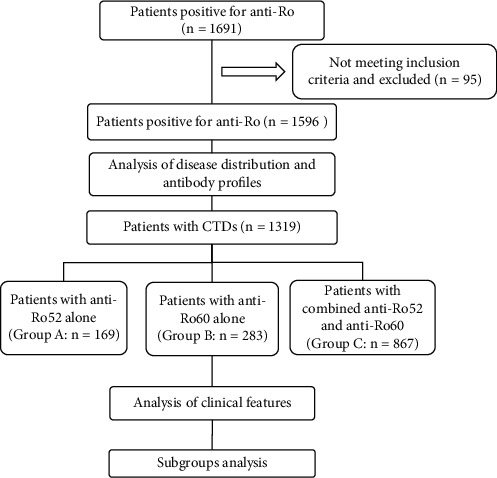
Flow chart of patient inclusion and grouping.

As shown in Figure 1, a total of 1596 patients who were positive for anti-Ro antibodies were included in the study, of which 1362 (85.3%) were female and the female-to-male ratio was 1362 : 234. The average age of the included patients was 45.5 ± 16.5 years. A total of 1340 (84.0%) patients were diagnosed with autoimmune diseases, of which 1319 (82.6%) were CTDs, with SLE (46.0%) and SS (19.0%) as the most common diseases. In addition, 256 patients (16.0%) were diagnosed with non-autoimmune diseases, which mainly comprised malignancies and infection.

Patients were divided into three subgroups according to the profiles of anti-Ro antibodies: Group A comprised patients who were positive for anti-Ro52 antibodies but negative for anti-Ro60 antibodies; Group B comprised patients who were positive for anti-Ro60 antibodies but negative for anti-Ro52 antibodies; and Group C comprised patients who were positive for both anti-Ro52 and anti-Ro60 antibodies. Compared with Groups B and C, patients were significantly older in Group A, with a statistically lower percentage of women (84.8% vs. 89.5% vs. 72.1%, *p* < 10^−4^) (Table 1). However, there was no significant difference in gender or age between Groups B and C.

### 3.2. Disease Distribution

The disease distributions of patients with anti-Ro antibodies are shown in Table 1. The predominance of autoimmune diseases in Group A was markedly less compared with that in Groups B and C (58.5% vs. 92.3% vs. 89.1% for Groups A, B, and C, respectively, *p* < 10^−4^). Patients in Group A were most likely to be diagnosed with CTDs such as IM (18.8%), SLE (17.6%), UCTD (17.6%), and SS (15.3%). In Group B, the most frequent diagnosis was SLE (47.6%), followed by UCTD (15.7%) and SS (12.9%), and in Group C, SLE (51.3%) and SS (21.6%) were the most common CTDs.

Compared with Group B, a significantly lower percentage of patients were diagnosed with SLE in Group A (47.6% vs. 17.6%, *p* < 10^−4^), while the proportion of patients with SS was significantly higher in Group C than that in Group B (12.9% vs. 21.6%, *p* < 10^−4^). Moreover, significantly higher proportions of patients with IM (18.8% vs. 1.0% vs. 2.5%, *p* < 10^−4^) and malignancies (30.4% vs. 8.3% vs. 21.5%, *p* = 0.044) were observed in Group A compared with those in Groups B and C. In addition, a significantly lower percentage of patients in Group C were diagnosed with RA compared with that of Group A (5.4% vs. 14.8%, *p* < 10^−4^) and Group B (5.4% vs. 11.5%, *p* = 0.008).

### 3.3. Comparison of Antibody Profiles

**Figure 2:**
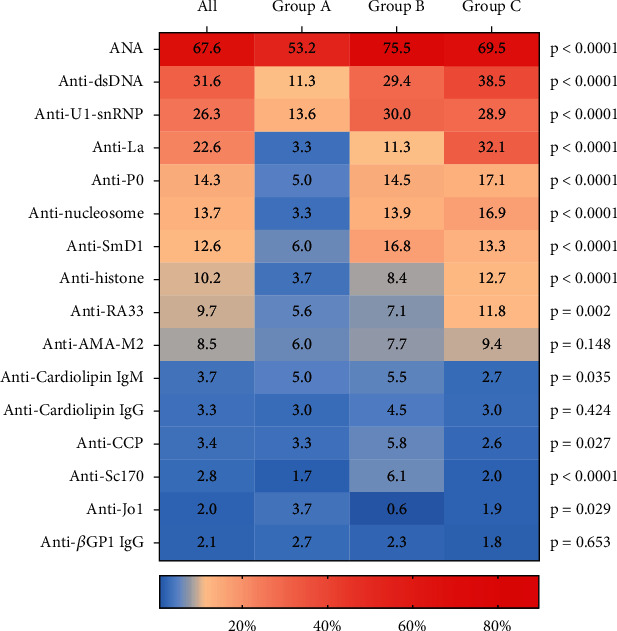
Comparison of autoantibody profiles in patients with positive anti-Ro antibodies.

A total of 1079 (67.6%) patients with anti-Ro antibodies were positive for ANA, and the most frequent coexistent antibodies were anti-dsDNA (31.6%), anti-U1-snRNP (26.3%), and anti-La (22.6%) (Figure 2).

In Group A, the proportions of some coexistent antibodies—including anti-dsDNA, anti-nucleosome, anti-histone, anti-La, anti-U1-snRNP, and anti-SmD1—were significantly lower compared with those in the other two groups. However, the highest positivity of anti-Jo1 antibodies was detected in Group A (3.7% vs. 0.6% vs. 1.9% for Groups A, B, and C, respectively, *p* = 0.029).

In Group B, the most common coexistent antibodies were anti-U1-snRNP (30.0%), anti-dsDNA (29.4%) and anti-SmD1 (16.8%). A total of 750 individuals (76.1%) in Group C had coexistent antibodies with the anti-Ro antibodies, and the most common were anti-dsDNA (38.5%), anti-La (32.1%), and anti-U1-snRNP (28.9%). The profiles of coexistent antibodies in Groups B and C were approximately similar, but the positivity of anti-La antibodies was significantly lower in Group B compared with that in Group C (11.3% vs. 32.1%, *p* < 10^−4^) (Supplementary Table 1).

### 3.4. Comparison of Laboratory Characteristics in Patients with Connective Tissue Diseases

Compared with Groups A and B, a significantly lower percentage of patients had an elevated level of CRP in Group C (41.4% vs. 28.4% for Groups A and C, respectively, *p* = 0.001; 36.0% vs. 28.4% for Groups B and C, respectively, *p* = 0.015) (Table 2). The prevalence of hypocomplementemia was significantly lower in Group A compared with that in the other groups (16.0% vs. 27.6% vs. 34.5% for Groups A, B, and C, respectively, *p* < 10^−4^), while the prevalence of hyperglobulinemia was significantly higher in Group C compared with that in Groups A and B (40.9% vs. 25.2% vs. 22.3%, *p* < 10^−4^).

### 3.5. Comparison of Clinical Features in Patients with Connective Tissue Diseases

As shown in Table 3 and Table 4, among the 1319 patients diagnosed with CTDs, the most frequent organ involvement was the musculoskeletal system (43.0%), followed by the hematological (40.5%) and mucocutaneous systems (31.5%). Accordingly, those patients with CTDs were characterized by arthralgia (38.8%), anemia (28.4%), and skin rash (26.5%). The prevalence of cardiovascular (14.0%), gastrointestinal (5.8%), and neuropsychiatric involvement (5.1%) was relatively lower compared with that of other organ involvement.

Musculoskeletal and pulmonary involvement was the most common organ involvements in Group A, with a prevalence of 48.5% and 45.0%, respectively. A significantly higher proportion of patients in Group A experienced pulmonary involvement compared with that in Groups B and C (45.0% vs. 24.0% vs. 23.1%, *p* < 10^−4^) (Table 4), with an accordingly higher prevalence of ILD (35.5% vs. 11.3% vs. 13.7%, *p* < 10^−4^) and PAH (10.1% vs. 5.3% vs. 3.6%, *p* = 0.001) in Group A (Table 3). However, compared with the other two groups, a significantly lower prevalence of renal, hematological, and neuropsychiatric involvements was observed in Group A (all *p* < 10^−4^), and proteinuria and hematuria rarely occurred in patients in Group A.

Patients in Group B predominantly had involvements of the musculoskeletal (62.5%), mucocutaneous (45.2%) and hematological (40.6%) systems, characterized by skin rash (35.7%), arthralgia (58.0%), and anemia (27.6%). Notably, the proportions of patients with musculoskeletal and mucocutaneous system involvements were significantly higher compared with those in Groups A and C (*p* < 10^−4^).

Compared with Group B, the proportion of patients with glandular involvement was significantly higher in Group C (28.6% vs. 19.1%, *p* = 0.003). Accordingly, a significantly higher proportion of patients suffered from xerostomia (26.4% vs. 16.6%, *p* = 0.002) and xerophthalmia (23.9% vs. 13.8%, *p* < 10^−4^) in Group C. In addition, a statistically lower prevalence of myalgia was detected in Group C versus Group B (4.4% vs. 9.5%, *p* = 0.002).

### 3.6. Subgroup Analyses

Subgroup analyses were further performed among patients with SLE, SS, and ILD (Table 5). In the SLE subgroup, a higher positivity of anti-cardiolipin IgG (16.1%), anti-cardiolipin IgM (12.9%), and anti-*β*GP1 (16.1%) was found in Group A compared with that in the other two groups. The prevalence of skin rash in Group B was significantly higher than that in Group A (48.5% vs. 19.4%, *p* = 0.003).

In the subgroup of patients with SS, compared with Group B, patients in Group C were more likely to experience xerophthalmia (60.0% vs. 24.3%, *p* < 10^−4^) and xerostomia (64.7% vs. 35.1%, *p* = 0.001). In addition, patients in Group C had the highest prevalence of hyperglobulinemia (39.5% vs. 0% vs. 16.2% for Groups C, A, and B, respectively, *p* < 10^−4^). Moreover, the highest incidences of ILD (37.0% vs. 2.7% vs. 15.8%, *p* = 0.001) and Raynaud's phenomenon (RP) (11.1% vs. 5.4% vs. 2.1%, *p* = 0.049) were observed in Group A compared with Groups B and C.

Patients with ILD were mainly diagnosed with SLE (24.2%), UCTD (21.8%), SS (19.4%), and IM (14.2%). For patients with ILD in Group A, the prevalence of SLE was significantly lower compared with that in the other groups (6.7% vs. 31.2% vs. 31.1% for Group A vs. Group B vs. Group C, respectively, *p* = 0.001), while the prevalence of IM was significantly higher than that in the other groups (33.3% vs. 0% vs. 8.4% for Group A vs. Group B vs. Group C, respectively, *p* < 10^−4^).

## 4. Discussion

Although there have been studies regarding the prevalence and clinical associations of anti-Ro antibodies, the results are not consistent [16, 17]. Therefore, we performed a large-scale study to investigate the clinical significance of anti-Ro antibodies. A significantly higher proportion of patients with isolated anti-Ro52 antibodies were diagnosed with IM and malignancies, and were also more likely to suffer from ILD and PAH compared with the patients with only isolated anti-Ro60 antibodies or those with both anti-Ro60 and anti-Ro52 antibodies. Compared with patients with isolated anti-Ro52 antibodies or anti-Ro60 antibodies, the positivity of anti-La antibodies was significantly higher in patients who were positive for both anti-Ro60 and anti-Ro52 antibodies, and these patients were also more likely to experience xerostomia and xerophthalmia, especially in individuals with SS. This study revealed distinct clinical features of patients with different profiles of anti-Ro antibodies, indicating the potential diagnostic and prognostic value of anti-Ro antibody profiles in clinical practice.

Ro60 and Ro52 are not part of a stable macromolecular complex and have different functions and clinical significance [36]. Ro60 is a clinically important target of autoantibodies in patients with rheumatic diseases, such as SS and SLE [5]. Anti-Ro60 antibodies were independently associated with a lower level of serum complement in patients with SLE [37]. Ro52 antigen, as an E3 ubiquitin ligase, was upregulated in peripheral blood mononuclear cells from patients with SLE or SS, which may increase the autoantigenic load in these patients [38]. In the current study, the most common CTDs in patients with both anti-Ro52 and anti-Ro60 antibodies were SLE and SS. The presence of isolated anti-Ro52 antibodies was more common in older men, which may be at least partially explain the higher prevalence of malignancies in the patients with anti-Ro52 alone. Moreover, patients with anti-Ro52 antibodies alone were significantly associated with IM, which is congruent with previous studies [9, 36].

In this study, a significantly lower percentage of patients with anti-Ro52 alone had other coexistent antibodies compared with the other two groups (anti-Ro60 alone and combined anti-Ro52 and anti-Ro60 antibodies), which may contribute to a correspondingly lower prevalence of CTDs in the isolated anti-Ro52 group. Anti-Ro52 antibody has long been recognized as one of myositis-associated autoantibodies, and often co-occurs with myositis-specific antibodies in patients with IM [9]. The frequency of anti-Ro52 antibodies was previously reported to be 58–74% in patients with anti-Jo1-positive IM [9, 39]. Accordingly, compared with patients with anti-Ro60, a significantly higher prevalence of anti-Jo1 antibodies was observed in patients with anti-Ro52 antibodies alone in the current study, which is consistent with a higher incidence of IM in the anti-Ro52 alone group [16]. Previous studies revealed a positive association between anti-CL and anti-Ro60 antibodies in patients with SLE [17]. However, in the present study, neither anti-CL nor anti-*β*GP1 antibodies were statistically related with the antibody profiles of anti-Ro60 and anti-Ro52. Further subgroup analysis did show that SLE patients with anti-Ro52 alone had the highest prevalence of anti-CL and anti-*β*GP1 antibodies among the three groups. However, a false positive result for antiphospholipid antibodies can be caused by infection [40], and in the present study, SLE patients with isolated anti-Ro52 were more likely to concurrently suffer from infection compared with the other groups, which may partially explain the highest prevalence of antiphospholipid antibodies in SLE patients with anti-Ro52 antibodies alone.

Anti-Ro52 was reported to be associated with a high prevalence and severity of ILD as well as a poor prognosis in patients with dermatomyositis [41], especially in anti-MDA5-positive patients [42]. Furthermore, patients with both anti-Ro52 and anti-Jo1 antibodies more frequently developed lung fibrosis and had more severe ILD compared with those with anti-Jo1 antibodies alone [43]. Data from the current study showed that the prevalence of pulmonary involvements, especially ILD, was increased in patients with anti-Ro52 antibodies alone compared with the other two groups (anti-Ro60 alone and combined anti-Ro52 and anti-Ro60 antibodies). This may be explained by the finding that a significantly higher proportion of patients with isolated anti-Ro52 were diagnosed with IM and had anti-Jo1 antibodies in this study. The association between anti-Ro52 and ILD has been reported in patients with various CTDs such as SS, SSc, and MCTD [10–12]. SSc, MCTD, and SLE are the most common causes of CTD-associated PAH [44]. A high incidence of micro- or macroangiopathy including digital tip ulcers, distal ischemia, and PAH was previously reported in CTD patients with anti-Ro52, including in patients with SSc [45]. Lee et al. [46] found that anti-Ro52 was independently associated with PAH and mortality in patients with SSc. Therefore, the higher prevalence of PAH in patients with isolated anti-Ro52 may be attributed to the higher prevalence of ILD and SSc. Notably, a higher prevalence of CTD patients with isolated anti-Ro52 suffered from RP. Subgroup analyses of patients with SS also showed that RP was significantly more frequent in patients with anti-Ro52 antibodies alone compared with the other two Ro antibody groups. The prevalence of lung involvement was previously reported to be significantly higher in SS patients with RP [47]. In the current study, a higher prevalence of ILD was observed in patients with SS who had anti-Ro52 antibodies alone, which may be attributed to a higher prevalence of RP in these patients.

Raúl et al. [48] found that xerophthalmia and xerostomia were positively associated with the antibody pattern of anti-Ro60 and anti-Ro52 as well as anti-La antibodies. Zampeli et al. [16] reported that patients with combined anti-Ro60 and anti-Ro52 had a higher frequency of sicca symptoms and salivary gland enlargement. A high titer of anti-Ro52 antibodies was associated with severe salivary dysfunction and a high level of gammaglobulin [8]. Moreover, patients with SS and concurrent anti-Ro and anti-La antibodies had more severe inflammatory infiltration of the salivary gland compared with those with anti-Ro alone [49]. Consistently, in the present study, patients with both anti-Ro52 and anti-Ro60 reactivity were more likely to experience xerophthalmia and xerostomia and also had a significantly higher anti-La positivity compared with the patients with isolated anti-Ro60 or anti-Ro52 positivity.

Anti-Ro60 antibodies are frequently detected in patients with SLE. Ruacho et al. [50] reported that SLE patients with anti-Ro60 antibodies had a higher prevalence of leukopenia and photosensitivity compared with those without anti-Ro60. Patients with SLE those are positive for anti-Ro60 antibodies are prone to be concurrently diagnosed with secondary SS [51], and also have a higher incidence of hypocomplementemia [37]. Congruent with these observations, patients with anti-Ro60 antibodies in the present study had a higher prevalence of hypocomplementemia and hyperglobulinemia.

There are some limitations to the current study. Sensitivity and specificity may vary significantly among different assays and kits. In addition, the immunoblot assay, as the most common method to detect anti-ENA in China, was used in this study. This further increases the difficulties in comparability with studies from Western countries, in which ELISA is the most frequent detection method for anti-ENAs. Furthermore, the study is a cross-sectional study from a single center. Multicenter longitudinal studies should be performed in the future to further investigate the clinical significance of different profiles of anti-Ro antibodies.

## 5. Conclusions

Disease phenotypes and clinical relevance may vary significantly in patients with different profiles of anti-Ro antibodies, indicating the potential diagnostic and prognostic value of anti-Ro antibodies in clinical practice. Patients with SS who are positive for both anti-Ro60 and anti- Ro52 antibodies are more likely to suffer from dryness of the mouth and eyes. IM and malignancies should be suspected in patients with solo anti-Ro52 antibodies. Furthermore, clinicians should also pay more attention to CTD patients with isolated anti-Ro52 antibodies due to a higher possibility of ILD and PAH in these patients. With the development of immunological detection technologies, automated quantitative assays should be adopted to replace the conventional assays to increase the comparability with studies from different countries. Additionally, multicenter longitudinal studies are required to further explore the clinical significance of anti-Ro antibody profiles.

## Figures and Tables

**Table 1:** Demographic characteristics and disease distribution of the included patients.

Parameters	Groups				*p* values			Overall
	All: *n* = 1596	Group A: Ro52 alone (*n* = 301)	Group B: Ro60 alone (*n* = 310)	Group C: Ro60 and Ro52 (*n* = 985)	Group A vs. group B	Group A vs. group C	Group B vs. group C	
Female (*n*, %)	1362 (85.3)	217 (72.1)	263 (84.8)	882 (89.5)	**<0.0001**	**<0.0001**	**0.024**	**<0.0001**
Age, years (mean ± SD)	45.5 ± 16.5	54.3 ± 17.1	43.4 ± 15.3	43.6 ± 15.8	**<0.0001**	**<0.0001**	0.975	**<0.0001**
AIDs (*n*, %)	1340 (84.0)	176 (58.5)	286 (92.3)	878 (89.1)	**<0.0001**	**<0.0001**	0.112	**<0.0001**
SLE	617 (46.0)	31 (17.6)	136 (47.6)	450 (51.3)	**<0.0001**	**<0.0001**	0.277	**<0.0001**
SS	254 (19.0)	27 (15.3)	37 (12.9)	190 (21.6)	0.468	0.059	**<0.0001**	**0.002**
RA	106 (7.90)	26 (14.8)	33 (11.5)	47 (5.4)	0.312	**<0.0001**	**0.008**	**<0.0001**
UCTD	195 (14.6)	31 (17.6)	45 (15.7)	119 (13.6)	0.597	0.159	0.357	0.308
IM	58 (4.3)	33 (18.8)	3 (1.0)	22 (2.5)	**<0.0001**	**<0.0001**	0.140	**<0.0001**
SV	12 (0.9)	2 (1.1)	7 (2.4)	3 (0.3)	0.322	0.161	**0.001**	**0.004**
SSc	30 (2.2)	8 (4.5)	11 (3.8)	11 (1.3)	0.713	**0.003**	**0.005**	**0.003**
APS	3 (0.2)	0 (0)	2 (0.7)	1 (0.1)	NA	NA	0.090	0.152
MCTD	4 (0.3)	1 (0.6)	1 (0.3)	2 (0.2)	0.728	0.439	0.724	0.740
Overlap syndrome	19 (1.4)	4 (2.3)	3 (1)	12 (1.4)	0.296	0.370	0.679	0.545
SpA	9 (0.7)	3 (1.7)	4 (1.4)	2 (0.2)	0.794	**0.009**	**0.016**	**0.022**
Organ-specific AIDs	21 (1.6)	7 (4.0)	3 (1.0)	11 (1.3)	**0.036**	**0.011**	0.784	**0.021**
RP	1 (0.1)	0 (0)	1 (0.3)	0 (0)	NA	NA	NA	NA
AOSD	2 (0.1)	0 (0)	0 (0)	2 (0.2)	NA	NA	NA	NA
ITP	9 (0.7)	3 (1.7)	0 (0)	6 (0.7)	NA	0.371	NA	0.054
Non-AIDs (*n*, %)	256 (16.0)	125 (41.5)	24 (7.7)	107 (10.9)	**<0.0001**	**<0.0001**	0.095	**<0.0001**
Malignancies^$^	63 (24.6)	38 (30.4)	2 (8.3)	23 (21.5)	**0.025**	0.125	0.138	**0.044**
Chronic diseases^∗^	66( 25.8)	38 (30.4)	2 (8.3)	26 (24.3)	**0.025**	0.300	0.085	0.069
Infections	50 (19.5)	24 (19.2)	5 (20.8)	21 (19.6)	0.853	0.935	0.893	0.983
Osteoarthritis	22 (8.6)	8 (6.4)	11 (45.8)	3 (2.8)	**<0.0001**	0.199	**<0.0001**	**<0.0001**
Gout	7 (2.7)	3 (2.4)	2 (8.3)	2 (1.9)	0.139	0.781	0.096	0.204
Gestation^#^	8 (3.1)	4 (3.2)	0 (0)	4 (3.7)	NA	0.823	NA	0.635
Others^†^	40 (15.6)	10 (8.0)	2 (8.3)	28 (26.2)	0.956	**<0.0001**	**<0.0001**	**<0.0001**

^$^Malignancies include lung cancer (*n* = 19), lymphoma (*n* = 10), as well as colorectal (*n* = 8), gastric (*n* = 6), breast (*n* = 8), ovarian (*n* = 6), prostate (*n* = 3), and nasopharyngeal (*n* = 3) cancers. ^∗^Chronic diseases include chronic obstructive pulmonary diseases, chronic kidney disease, chronic heart failure, sequelae of cerebral infarction, hypertension, and diabetes mellitus. ^#^Gestation includes preeclampsia, eclampsia, recurrent spontaneous abortion, and gestational hypertension. ^†^Others include acute myocardial infarction, acute cerebral hemorrhage, anemia, arrhythmia, etc. AIDs: autoimmune diseases; SLE: systemic lupus erythematosus; SS: primary Sjögren's syndrome; RA: rheumatoid arthritis; UCTD: undifferentiated connective tissue disease; IM: inflammatory myositis; SV: systemic vasculitis; SSc: systemic sclerosis; APS: antiphospholipid syndrome; SpA: spondylarthritis; MCTD: mixed connective tissue disease; RP: relapsing polychondritis; AOSD: adult-onset Still's disease; ITP: immune thrombocytopenic purpura; NA: not applicable.

**Table 2:** Comparison of laboratory data in patients with connective tissue diseases.

Parameters (*n*, %)	Groups				*p* values			Overall
	All *n* = 1319	Group A: Ro52 alone *n* = 169	Group B: Ro60 alone *n* = 283	Group C: Ro60 and Ro52 *n* = 867	Group A vs. group B	Group A vs. group C	Group B vs. group C	
IgG^#^>18 g/L	420 (34.9)	38 (25.2)	58 (22.3)	324 (40.9)	0.509	**<0.0001**	**<0.0001**	**<0.0001**
C3<0.8 g/L	404 (30.6)	27 (16.0)	78 (27.6)	299 (34.5)	**0.005**	**<0.0001**	**0.031**	**<0.0001**
C4<0.15 g/L	502 (38.1)	43 (25.4)	92 (32.5)	367 (42.3)	0.112	**<0.0001**	**0.003**	**<0.0001**
CRP>5 mg/L	418 (31.7)	70 (41.4)	102 (36.0)	246 (28.4)	0.255	**0.001**	**0.015**	**0.001**
ESR>20 mm/h	754 (57.2)	92 (54.4)	145 (51.2)	517 (59.6)	0.510	0.210	**0.013**	**0.035**
eGFR<60 mL/min/1.73m^2^	108 (8.2)	18 (10.7)	21 (7.4)	69 (8.0)	0.237	0.248	0.770	0.439
Albumin<30 g/L	218 (16.5)	25 (14.8)	42 (14.8)	151 (17.4)	0.989	0.406	0.314	0.485
RF^∗^	208 (20.1)	35 (28.2)	42 (17.7)	131 (19.4)	**0.021**	**0.026**	0.575	**0.046**

CRP: C-reaction protein; ESR: erythrocyte sedimentation rate. ^#^116 patients without serum IgG result. ^∗^A total of 282 patients did not have rheumatoid factor tested.

**Table 3:** Comparison of clinical features in patients with connective tissue diseases.

Parameters (*n*, %)	Groups				*p* values			Overall
	All: *n* = 1319	Group A: Ro52 alone *n* = 169	Group B: Ro60 alone *n* = 283	Group C: Ro60 and Ro52 *n* = 867	Group A vs. group B	Group A vs. group C	Group B vs. group C	
Disease duration, years (mean ± SD)	5.3 ± 6.7	3.5 ± 5.3	5.3 ± 7.5	5.6 ± 6.6	**0.019**	**<0.0001**	0.063	**<0.0001**
Skin rash	349 (26.5)	40 (23.7)	101 (35.7)	208 (24.0)	**0.008**	0.928	**<0.0001**	**<0.0001**
Alopecia	99 (7.5)	6 (3.6)	37 (13.1)	56 (6.5)	**0.001**	0.145	**<0.0001**	**<0.0001**
Oral ulcer	55 (4.2)	6 (3.6)	16 (5.7)	33 (3.8)	0.315	0.873	0.182	0.366
Arthralgia	512 (38.8)	72 (42.6)	164 (58.0)	276 (31.8)	**0.002**	**0.007**	**<0.0001**	**<0.0001**
Myalgia	80 (6.1)	16 (9.5)	26 (9.2)	38 (4.4)	0.921	**0.007**	**0.002**	**0.002**
Xerophthalmia	277 (21.0)	31 (18.3)	39 (13.8)	207 (23.9)	0.160	0.118	**<0.0001**	**0.001**
Xerostomia	310 (23.5)	34 (20.1)	47 (16.6)	229 (26.4)	0.346	0.085	**0.001**	**0.002**
Anemia	375 (28.4)	39 (23.1)	78 (27.6)	258 (29.8)	0.292	**0.079**	0.481	0.198
Leukocytopenia	295 (22.4)	28 (16.6)	61 (21.6)	20 6(23.8)	0.197	**0.041**	0.446	0.114
Thrombocytopenia	128 (9.7)	11 (6.5)	27 (9.5)	90 (10.4)	0.261	0.121	0.685	0.297
Proteinuria	208 (15.8)	10 (5.9)	53 (18.7)	145 (16.7)	**<0.0001**	**<0.0001**	0.438	**0.001**
Hematuria	99 (7.5)	3 (1.8)	29 (10.2)	67 (7.7)	**0.001**	**0.005**	0.183	**0.004**
ILD	211 (16.0)	60 (35.5)	32 (11.3)	119 (13.7)	**<0.0001**	**<0.0001**	0.296	**<0.0001**
PAH	63 (4.8)	17 (10.1)	15 (5.3)	31 (3.6)	0.056	**<0.0001**	0.199	**0.001**
RP	88 (6.7)	18 (10.7)	22 (7.8)	48 (5.5)	0.297	**0.013**	0.172	**0.036**

ILD: interstitial lung disease; PAH: pulmonary arterial hypertension; RP: Raynaud's phenomenon.

**Table 4:** Comparison of organ involvement in patients with connective tissue diseases.

Parameters (*n*, %)	Groups				*p* values			Overall
	All *n* = 1319	Group A: Ro52 alone *n* = 169	Group B: Ro60 alone *n* = 283	Group C: Ro60 and Ro52 *n* = 867	Group A vs. group B	Group A vs. group C	Group B vs. group C	
Musculoskeletal	567 (43.0)	82 (48.5)	177 (62.5)	308 (35.5)	**0.004**	**0.001**	**<0.0001**	**<0.0001**
Hematological	534 (40.5)	50 (29.6)	115 (40.6)	369 (42.6)	**0.018**	**0.002**	0.569	**0.007**
Mucocutaneous	415 (31.5)	43 (25.4)	128 (45.2)	244 (28.1)	**<0.0001**	0.473	**<0.0001**	**<0.0001**
Glandular	339 (25.7)	37 (21.9)	54 (19.1)	248 (28.6)	0.471	0.074	**0.002**	**0.003**
Pulmonary	344 (26.1)	76 (45.0)	68 (24.0)	200 (23.1)	**<0.0001**	**<0.0001**	0.740	**<0.0001**
Ocular	337 (25.5)	37 (21.9)	58 (20.5)	242 (27.9)	0.724	0.107	**0.014**	**0.023**
Renal	263 (19.9)	12 (7.1)	71 (25.1)	180 (20.8)	**<0.0001**	**<0.0001**	0.126	**<0.0001**
Cardiovascular	185 (14.0)	23 (13.6)	48 (17.0)	114 (13.1)	0.343	0.872	0.109	0.280
Gastrointestinal	77 (5.8)	7 (4.1)	20 (7.1)	50 (5.8)	0.204	0.397	0.427	0.439
Neuropsychiatric	67 (5.1)	4 (2.4)	24 (8.5)	39(4.5)	**0.009**	0.204	**0.011**	**0.007**

**Table 5:** Subgroup analyses of patients with different connective tissue diseases.

Subgroups	Parameters (*n*, %)	Groups			*p* values			Overall
		Group A: Ro52 alone *n* = 169	Group B: Ro60 alone *n* = 283	Group C: Ro60 and Ro52 *n* = 867	Group A vs. group B	Group A vs. group C	Group B vs. group C	
SLE	All	31 (18.3)	136 (48.1)	450 (51.9)	**<0.0001**	**<0.0001**	0.261	**<0.0001**
	Skin rash	6 (19.4)	66 (48.5)	134 (29.8)	**0.003**	0.217	**<0.0001**	**<0.0001**
	Arthralgia	10 (32.3)	82 (60.3)	137 (30.4)	**0.005**	0.832	**<0.0001**	**<0.0001**
	Myalgia	1 (3.2)	16 (11.8)	12 (2.7)	0.276	1.000	**<0.0001**	**<0.0001**
	IgG>18 g/L^∗^	15 (53.6)	28 (22.2)	149 (35.5)	**0.001**	0.054	**0.005**	**0.002**
	ANA	19 (61.3)	107 (78.7)	272 (60.4)	**0.042**	0.926	**<0.0001**	**<0.0001**
	ACA-IgG	5 (16.1)	10 (7.4)	21 (4.7)	0.232	**0.020**	0.220	**0.022**
	ACA-IgM	4 (12.9)	12 (8.8)	17 (3.8)	0.720	0.051	**0.017**	**0.011**
	Anti-*β*GP1	5 (16.1)	4 (2.9)	14 (3.1)	**0.013**	**0.002**	1.000	**0.001**
	Anti-ds-DNA	17 (54.8)	67 (49.3)	285 (63.3)	0.575	0.344	**0.003**	**0.011**
	Anti-La	1 (3.2)	21 (15.4)	142 (31.6)	0.128	**0.001**	**<0.0001**	**<0.0001**
	Infection	11 (35.5)	42 (30.9)	101 (22.4)	0.619	0.097	**0.045**	0.052

SS	All	27 (16.0)	37 (13.1)	190 (21.9)	0.392	0.083	**0.001**	**0.002**
	Xerophthalmia	12 (44.4)	9 (24.3)	114 (60.0)	0.090	0.125	**<0.0001**	**<0.0001**
	Xerostomia	12 (44.4)	13 (35.1)	123 (64.7)	0.451	**0.042**	**0.001**	**0.001**
	Arthralgia	11 (40.7)	25 (67.6)	51(26.8)	**0.033**	0.135	**<0.0001**	**<0.0001**
	RP	3 (11.1)	2 (5.4)	4 (2.1)	0.713	**0.013**	0.559	**0.049**
	ILD	10 (37.0)	1 (2.7)	30 (15.8)	**0.001**	**0.008**	**0.034**	**0.001**
	IgG>18 g/L	0 (0)	6 (16.2)	75 (39.5)	0.096	**<0.0001**	**<0.0001**	**<0.0001**
	ANA	12 (44.4)	23 (62.2)	150 (78.9)	0.160	**<0.0001**	**0.028**	**<0.0001**
	Anti-La	3 (11.1)	3 (8.1)	91( 47.9)	1.000	**<0.0001**	**<0.0001**	**<0.0001**

ILD	All	60 (35.5)	32 (11.3)	119 (13.7)	**<0.0001**	**<0.0001**	0.296	**<0.0001**
	SLE	4 (6.7)	10 (31.2)	37 (31.1)	**0.004**	**<0.0001**	0.986	**0.001**
	UCTD	15 (25.0)	12 (37.5)	19(16.0)	0.193	0.162	**0.007**	**0.026**
	pSS	10 (16.7)	1 (3.1)	30 (25.2)	0.122	0.178	**0.006**	**0.015**
	IM	20 (33.3)	0 (0)	10 (8.4)	**<0.0001**	**<0.0001**	0.195	**<0.0001**

SLE: systemic lupus erythematosus; pSS: primary Sjögren's syndrome; SSc: systemic sclerosis; ANA: anti-nuclear antibodies; ACA-IgG: Anti-cardiolipin antibodies IgG; ACA-IgM: Anti-cardiolipin antibodies IgM; Anti-*β*GP1: Anti-*β*2-glycoprotein antibodies; Anti-ds-DNA: anti-double-stranded DNA antibodies; RP: Raynaud's phenomenon; ILD: interstitial lung disease. ^∗^A total of 42 patients with SLE did not have serum IgG tested.

## Data Availability

The data that support the findings of this study are available upon request.

## Supplementary Materials

Supplementary Materials Comparison of autoantibody profiles in patients with positive anti-Ro antibodies (including *p* values between groups) is shown in Supplementary Table 1.
