# Supplementary material for: Clinical Significance of Different Profiles of anti-Ro Antibodies in Connective Tissue Diseases
Source: J Immunol Res. 2023 Jan 25;2023:9195157. doi: 10.1155/2023/9195157 (PMC9891828; doi:10.1155/2023/9195157)
Supplement: Supplementary Materials — Comparison of autoantibody profiles in patients with positive anti-Ro antibodies (including p values between groups) is shown in Supplementary Table 1. [file 9195157.f1.docx]

**Supplementary TABLE 1** **Comparison of autoantibody profiles in patients with positive anti-Ro antibodies**

| **Parameters (n, %)** | **Groups** | | | | **p values** | | |  |
| --- | --- | --- | --- | --- | --- | --- | --- | --- |
|  | **All:**  **n=1596** | **Group A: Ro52 alone**  **(n=301)** | **Group B: Ro60 alone**  **(n=310)** | **Group C: Ro60 and Ro52**  **(n=985)** | **Group A vs. group B** | **Group A vs. group C** | **Group B vs. group C** | **Overall** |
| ANA | 1079(67.6) | 160(53.2) | 234(75.5) | 685(69.5) | **＜0.0001** | **＜0.0001** | **0.044** | **＜0.0001** |
| Anti-dsDNA | 504(31.6) | 34(11.3) | 91(29.4) | 379(38.5) | **＜0.0001** | **＜0.0001** | **0.004** | **＜0.0001** |
| Anti-U1-snRNP | 419(26.3) | 41(13.6) | 93(30.0) | 285(28.9) | **＜0.0001** | **＜0.0001** | 0.719 | **＜0.0001** |
| Anti-La | 361(22.6) | 10(3.3) | 35(11.3) | 316(32.1) | **＜0.0001** | **＜0.0001** | **＜0.0001** | **＜0.0001** |
| Anti-P0 | 228(14.3) | 15(5.0) | 45(14.5) | 168(17.1) | **＜0.0001** | **＜0.0001** | 0.293 | **＜0.0001** |
| Anti-nucleosome | 219(13.7) | 10(3.3) | 43(13.9) | 166(16.9) | **＜0.0001** | **＜0.0001** | 0.213 | **＜0.0001** |
| Anti-SmD1 | 201(12.6) | 18(6.0) | 52(16.8) | 131(13.3) | **＜0.0001** | **0.001** | 0.126 | **＜0.0001** |
| Anti-histone | 162(10.2) | 11(3.7) | 26(8.4) | 125(12.7) | **0.014** | **＜0.0001** | **0.040** | **＜0.0001** |
| Anti-RA33 IgG | 155(9.7) | 17(5.6) | 22(7.1) | 116(11.8) | 0.464 | **0.002** | **0.020** | **0.002** |
| Anti-AMA-M2 | 135(8.5) | 18(6.0) | 24(7.7) | 93(9.4) | 0.389 | 0.061 | 0.363 | 0.148 |
| Anti-cardiolipin IgM | 59(3.7） | 15(5.0) | 17(5.5) | 27(2.7) | 0.781 | 0.055 | **0.020** | **0.035** |
| Anti-cardiolipin IgG | 53(3.3） | 9(3.0） | 14(4.5) | 30(3.0) | 0.322 | 0.961 | 0.213 | 0.424 |
| Anti-CCP | 54(3.4） | 10(3.3) | 18(5.8) | 26(2.6) | 0.142 | 0.530 | **0.007** | **0.027** |
| Anti-Scl70 | 44(2.8) | 5(1.7) | 19(6.1) | 20(2.0) | **0.004** | 0.685 | **＜0.0001** | **＜0.0001** |
| Anti-Jo1 | 32(2.0) | 11(3.7) | 2(0.6) | 19(1.9) | **0.010** | 0.083 | 0.119 | **0.029** |
| Anti-βGP1 IgG | 33(2.1) | 8(2.7) | 7(2.3) | 18(1.8) | 0.750 | 0.370 | 0.631 | 0.653 |

ANA: anti-nuclear antibodies; Anti-dsDNA: anti-double-stranded DNA antibodies; Anti-U1-snRNP: anti-U1-small nuclear ribonucleoprotein antibodies; Anti-RA33: Anti-rheumatoid arthritis 33 antibodies; Anti-βGP1: Anti-β2-glycoprotein antibodies; Anti-AMA-M2: anti-mitochondrial antibody M2 subtype antibodies; RF: Rheumatoid factor; Anti-CCP: Anti-cyclic citrullinated peptide antibodies
